# Barriers to Colorectal Cancer Screening in Pakistan

**DOI:** 10.7759/cureus.1477

**Published:** 2017-07-16

**Authors:** Fariha Hasan, Sayed Mustafa Mahmood Shah, Misbah Munaf, Muhammad R Khan, Shayan Marsia, Syed Muhammad Haaris, Muhammad Hammad Shaikh, Ismail Abdur Rahim, Muhammad Salar Anwar, Kassam S Qureshi, Maham Iqbal, Sara Qazi, Burhanuddin A Kasi, Mahnoor Tahir, Syed Inam Ur Rehman, Kaneez Fatima

**Affiliations:** 1 Department of Internal Medicne, Dow University of Health Sciences (DUHS), Karachi, Pakistan; 2 Department of Internal Medicine, Dow University of Health Sciences (DUHS), Karachi, Pakistan; 3 Deparrment of Internal Medicine, Dow University of Health Sciences (DUHS), Karachi, Pakistan; 4 Department of Internal Medicine, Jinnah Medical and Dental College

**Keywords:** colorectal cancer, crc, crc public awareness, screening, barriers

## Abstract

Background

The prevalence of colorectal cancer (CRC) is growing in Pakistan; however, there are no national screening programs or guidelines in place to curb its development. This study was conducted with the aim of ascertaining public awareness and attitudes regarding CRC and current screening practices. Furthermore, the study assessed perceived barriers which could impact future screening processes.

Methods

A cross-sectional, questionnaire-based study was conducted among urban dwellers of Karachi, Pakistan. We excluded any individuals belonging to the medical profession, those diagnosed previously with CRC or having any significant co-morbidity. The validated and pre-tested questionnaire was administered among the study participants to record demographic information, awareness of CRC risk factors, symptoms and screening tests. Attitudes towards screening and perceived barriers to screening were also assessed. Data were analyzed using Statistical Package for Social Sciences (SPSS version 20.0) (IBM Corp., Armonk, NY). A knowledge score, out of a total of 14 points was calculated to reflect a participant’s overall knowledge regarding CRC risk factors and signs/symptoms.

Results

The prevalence of CRC screening in eligible individuals (50 years or older) was 2.6% in our study population. Positive attitudes towards CRC management and screening were observed, with 75.1% (n = 296) acknowledging the preventive role of screening tests. Despite this only 14.9% (n = 58) of study participants expressed a future desire to undergo screening. Major barriers to screening were reported to be “a lack of knowledge regarding the screening procedure”, a “lack of screening facilities” and that the “screening procedure is too expensive”. A majority (n = 285, 72.3%) of the participants expressed a greater willingness to undergo screening if their doctor recommended it.

Conclusion

A national CRC screening and awareness program should be launched to promote awareness and facilitate screening in risk groups. General practitioners are needed to play a key role in counseling patients and endorsing healthy screening practices.

## Introduction

Pakistan, like all other South Asian countries, is placed in a low-risk zone for colorectal cancer (CRC). However, recent studies have reported a surge in CRC cases for patients above the age of 50 years in Pakistan [1]. Elevated risk of CRC incidence is linked to high intake of preserved eatables, animal food, smoking, heavy alcohol consumption and inflammatory bowel disease. Declined physical activity levels accompanied by substantial rise in prevalence of obesity are also major contributors to the risk factors [2]. These risk factors are extensively outspread in Pakistani society. Hence, over the next few decades it is projected that there will be a rapid rise in CRC cases [1].

The US Preventative Service Task Force (USPSTF) recommends that options for CRC screening include colonoscopy every 10 years, sigmoidoscopy every 5 years and an annual Fecal Occult Blood Test (FOBT) or Fecal Immunochemical Test (FIT) beginning at age 50 and continuing until age 75 [3]. However, similar professional and national health guidelines are severely lacking in both rural and urban areas of Pakistan, producing a lack of awareness regarding CRC and unfamiliarity with the avenues of CRC screening.

Adherence to CRC screening has been advocated as an effective strategy in detection of pre-cancerous polyps in asymptomatic patients [4-5]. In Pakistan, the paucity of awareness is a key barrier to screening. Alternately, logistic and financial considerations, a lack of screening facilities, cultural norms or myths regarding screening procedures and religious beliefs can all serve as important barriers to successful CRC screening [6]. The present literature does not provide sufficient data regarding the public awareness of CRC in Pakistan, prevalence of current screening practices, public attitudes towards screening and the health implications arising thereof.

Accordingly, the primary objectives of this study were to ascertain the knowledge regarding risk factors and symptoms of CRC, and to determine the understanding and prevalence of CRC screening methods. Our secondary objective was to investigate key determinants of poor compliance to CRC screening and to evaluate the barriers to effective uptake of screening in a Pakistani population.

## Materials and methods

A cross-sectional survey was conducted in the city of Karachi from 25th December 2016 till 31st January 2017. A sample size of 384 was calculated using Open Epi, under an anticipated frequency of 50% and 95% confidence interval. A total sample of 450 urban dwellers was surveyed across the various municipalities of the city. Eligible study participants included all urban dwellers of Karachi above the age of 18 years. We excluded any individuals not permanently residing in the metropolis, those belonging to the medical profession, those diagnosed previously with CRC or having any significant co-morbidity such as heart disease, cancer or any such chronic disease. We did not account for ethnic heterogeneity, contrary to other studies [7-8] of the same nature because Karachi is a city of wide ethnic diversity.

A standardized 19 item questionnaire was designed following an extensive literature review and was translated into Urdu; the native language. The questionnaire was adapted from similar studies conducted in Malaysia [9] and Saudi Arabia [10], as no such study has been performed in Pakistan before. Following a thorough vetting of the questionnaire by medical professional and experts in the fields of epidemiology; a pilot study was conducted on a convenient sample of 30 individuals to assess its validity and clarity. The questionnaire and translation were further refined based on results of the pilot study.

The questionnaire comprised of four sections. The first section recorded basic demographic details while the second section assessed a participants’ knowledge and perceptions regarding the CRC risk factors, signs and symptoms and screening modalities. Screening practices and perceived barriers towards screening were assessed by the third and fourth sections, respectively. Screening was defined as a history of testing for CRC using a standardized test such as colonoscopy, sigmoidoscopy and FOBT or FIT.

Data collection was initiated using the pre-tested questionnaire. Informed, written consent was taken from each willing study participant. Participants who could not read or write were interviewed under a standard operating protocol by experienced researchers. Incompletely filled forms and those from the pilot study were excluded from the final analysis.

Data were analyzed using Statistical Package for Social Sciences (SPSS version 20.0) (IBM Corp., Armonk, NY). Categorical variables were expressed using frequencies and percentages. Continuous variables were presented as a mean and standard deviation. A knowledge score was calculated to reflect a participant's overall knowledge regarding CRC risk factors and signs/symptoms. It was scored out of a total of 14 points. Chi-squared test with 95% confidence interval was used to compare categorical variables. Differences in continuous variables, such as knowledge score, with respect to categorical variables, were assessed using independent T-test. A 5% level of significance was used throughout the study.

## Results

Of the 450 study participants who were approached, 400 consented to participate in this study, thus yielding a response rate of 88.9%. The basic demographic characteristics of study participants are presented in Table 1.

**Table 1:** Socio-demographic characteristics of study population.

Socio-demographic characteristics		Percentages (%)
Gender	Male	57.6
	Female	42.4
Age groups	Youth (less than 24 years)	7.8
	Adults (25 to 64 years)	86.3
	Seniors (65 years and over)	5.9
Marital status	Married	80.1
	Unmarried	19.9
Employment status	Employed full-time	53.9
	Employed part-time	13.9
	Unemployed	25.0
	Retired	7.2
Highest level of education	University	67.8
	Secondary school (6th-12th grade)	22.1
	Primary school (1st-5th grade)	5.8
	No formal education	4.3
Socio-economic class	Upper	21.7
	Middle	70.1
	Lower	8.2

The knowledge of participants regarding CRC risk factors and symptoms was assessed using a standardized knowledge score. The mean knowledge score was 7.52 +/- 3.36 out of a total of 14 (Table 2).

**Table 2:** Overall knowledge scores of study participants, and scores stratified by demographic characteristics.

Demographic characteristics	Knowledge score (Mean +/- SD)	p-value
Overall	7.52 +/- 3.36	
Males	7.00 +/- 3.35	0.001
Females	8.10 +/- 3.27	
Youth	7.70 +/- 3.19	0.856
Adults	7.57 +/- 3.38	
Seniors	7.96 +/- 3.02	
Married	7.82 +/- 3.30	0.319
Unmarried	7.39 +/- 3.07	
Employed full-time	7.43 +/- 3.41	0.612
Employed part-time	8.02 +/- 3.30	
Unemployed	7.81 +/- 3.21	
Retired	7.78 +/- 2.98	

Females had greater knowledge than males (p = 0.001). The most commonly recognized symptom for CRC was rectal bleeding (n = 259, 67.1%). No significant differences in knowledge score were observed across age groups, marital status or employment status (p > 0.05). Up to 43.8% (n = 175) of the study participants were aware of at least one of the three CRC screening tests; with colonoscopy being the most recognized screening option (n = 146, 37.9%), followed by FOBT (n = 101, 26.3%) and sigmoidoscopy (n = 71, 18.8%).

Perceptions of participants towards CRC was assessed and found to be significantly associated with knowledge score (Table 3). Participants who perceived CRC as a disease which may “present without symptoms”, a “common disease”, a “fatal disease” or a “cure-able disease” were more likely to demonstrate superior knowledge (p < 0.001). An acknowledgement of the therapeutic role of screening in detecting CRC at a stage “when treatment is most effective” was acknowledged by approximately three-quarters (n = 296, 75.1%) of study participants, and was associated with greater CRC knowledge scores (p < 0.001) (Table 3).

**Table 3:** Knowledge scores stratified by colorectal cancer (CRC) perceptions.

Perception questions	Response (%)		Knowledge score (Mean +/- SD)		p-value
	Yes	No/I don’t know	Yes	No/I don’t know	
Can CRC present without symptoms?	40.3	59.7	8.56 +/- 3.16	6.89 +/- 3.28	0.000
Is CRC a common disease?	22.6	77.4	9.15 +/- 3.29	7.11 +/- 3.19	0.000
Is CRC a fatal disease?	71.1	28.9	8.15 +/- 3.13	6.17 +/- 3.37	0.000
Is CRC curable?	57.7	42.3	8.09 +/- 3.26	6.84 +/- 3.29	0.000
Can screening enable timely and effective treatment of CRC?	75.1	24.9	7.91 +/- 3.21	6.30 +/- 3.43	0.000

The prevalence of prior CRC screening in individuals 50 years and older was 2.6% (n = 4). Almost 4% of study participants (n = 18) stated that their relatives had been screened for the disease (Table 4).

**Table 4:** Prevalence of colorectal cancer (CRC) screening practices and their association with knowledge scores.

History of CRC or screening	Response (%)	Knowledge score (Mean +/- SD)	p-value
Have any relatives been diagnosed with CRC?	Yes (4.8)	8.68 +/- 2.60	0.122
	No (95.2)	7.46 +/- 3.38	
Have any relatives been screened for CRC?	Yes (4.6)	10.2 +/- 2.26	0.001
	No (95.4)	7.40 +/- 3.31	
Have you ever been screened for CRC (for individuals >50 years)?	Yes (2.6)	11.0 +/- 1.63	0.058
	No (97.4)	7.67 +/- 3.48	
Do you intend to get screened for CRC in the future?	Yes (14.9)	7.91 +/- 3.37	0.362
	No (85.1)	7.48 +/- 3.32	

A similar percentage (4.8%, n = 19) reported a CRC diagnosis in relatives. It was found that those reporting a history of CRC diagnosis in relatives, a screening history in relatives and a screening history for themselves had greater knowledge than those who reported no such history. However, this difference was significant in those who reported a history of CRC screening in relatives (p = 0.001) (Table 4). Only 14.9% (n = 58) of participants expressed a future intention for CRC screening, which was not significantly associated with knowledge scores (p = 0.335). Factors predictive of a greater likelihood to undergo future CRC screening included being male (p = 0.032), adult age group (p = 0.046) and a university level education (p = 0.012).

Table 5 illustrates the responses to attitude statements regarding CRC screening.

**Table 5:** Attitude statements of study participants. CRC: Colorectal cancer.

Attitude statement	Response		
	Agree (%)	Neutral (%)	Disagree (%)
The screening procedure is not safe.	22.1	38.0	39.8
The screening procedure is not effective.	16.4	33.3	50.3
I am afraid of getting CRC in the future.	40.4	28.6	31.0
I am afraid of receiving abnormal results if screened.	41.2	24.3	34.5
I would feel embarrassed if I got screened.	27.3	21.4	51.3
There is a lack of screening facilities.	50.5	37.0	12.5
My lack of knowledge regarding CRC presents a barrier to screening.	61.5	23.2	15.3
If I am meant to get CRC, I will, irrespective of screening.	46.8	30.7	22.5
My friends and family are in favor of my getting screened.	40.7	36.1	23.3
I do not have time to get screened.	28.5	27.9	43.6
I would be more likely to have a colonoscopy if my Dr. told me to.	73.1	19.0	7.9
Screening is too expensive.	51.3	38.7	10.0

Most of the study participants acknowledged a greater likelihood of CRC screening if it was recommended by their doctor. A significant proportion agreed that their “lack of knowledge regarding the screening procedure” was a barrier for screening or felt the screening procedure is “too expensive”. An expression that the “screening procedure is not effective” or a feeling of “embarrassment if I was screened” was the least agreed to statements. Subgroup analysis showed that participants with greater knowledge scores were less likely to believe that the “screening procedure is not safe” (p < 0.001) or “not effective” (p = 0.002).

## Discussion

We found that the general awareness among the population regarding the symptoms and risk factors about CRC is poor, which is in alignment with some of the surveys conducted by Javanparast, et al. [11], Wong, et al. [12], Imran, et al. [13] and Harewod, et al. [14] in various parts of the world. But this result was contradictory to the findings of the study conducted by Suan, et al. in Malaysia [9]. This is not surprising given that the Pakistani population, according to previous studies, is reported to have poor knowledge regarding cancer [15]. Additionally, we found that females displayed greater knowledge than males, not only about the disease but also available screening procedures such as FOBT, sigmoidoscopy, and colonoscopy. Scoring differences between males and females may be due to the latter participating in cancer prevention programs, particularly breast and cervical cancers [16], making women more aware of various malignancies. This, in turn, emphasizes on the integral role of Primary Care Providers (PCP), and makes it their responsibility to educate high-risk individuals, i.e., older males on the importance of screening, and to promote healthy lifestyle practices among younger age groups [17-18]. This is further strengthened by more female participants in the study than males, agreeing to get screened if their “primary care provider recommends it” (p < 0.005), and suggests that adherence to screening practices is possible with a little encouragement.

Relationships between older age groups and knowledge of screening modalities have been suggested [19], however, our data proposes that age is not related to this factor. Moreover, one of the key findings of our study was the identification of rectal bleeding as the most commonly known symptom (67.1%) among the population which is in line with previous studies [9, 20]. This result is not surprising as rectal bleeding is the most commonly associated symptom of CRC [21-22]. Our study also made a notable observation regarding a participant's perception of CRC as a “fatal” (71.1%) and “curable” (57.7%) disease. This indicates that the people are conscious about the severity of the disease. Such participants displayed superior knowledge about the symptoms and risk factors, as opposed to those who perceived otherwise.

Approximately three quarters (75.1%) of the population were aware of the matter that timely screening practices enable effective treatment of CRC. This shows that the population is aware of the importance of screening procedures and its implication upon the ultimate survival and management of the patient. Up to 43.8% (n = 175) of the sample population was aware of any one of the three CRC screening tests, with colonoscopy being the most well-known (37.9%). What is disappointing about these findings is that even with good awareness only 14.5% participants intended to undergo future screening. When screening practices of individuals 50 years and older, which is the recommended age for initiation of screening, were assessed, the prevalence of CRC screening was found to be a mere 2.6%. This is comparable to a study conducted in Spain where the prevalence of CRC is much higher [23]. The poor adherence to CRC screening may be attributed to multiple factors that may influence the health-seeking behavior of participants. Of these, the tendency to brush off possible cancer-related symptoms, due to a lack of knowledge, as being insignificant or transient has been reported by Simon, et al. [16]. While our results did not support a direct association between CRC knowledge scores and future screening intentions, we did observe that those reporting greater screening intentions were more likely to perceive a fear for acquiring CRC in the future. This suggests that a heightened state of cancer-vigilance may have greater impacts on screening practices than a mere recollection of disease symptoms. The same is reinforced by Simon, et al.’s findings, whereby higher Cancer Awareness Measure symptom knowledge scores were not predictive of health-seeking behaviors in the United Kingdom population [16].

Our study evaluated the influence of various social factors on screening practices. A lack of knowledge regarding the risk factors associated with CRC, nature of screening procedures and the expenses involved constituted the major obstacles to effective uptake of screening. Earlier literature on this subject has already established an association between awareness and the willingness to screen [26], and our findings are consistent. Contrary to two studies discussing social support from friends and family as an important factor towards adherence to CRC screening [24-25], our findings did not strongly reflect this proposition. This is surprising as Pakistan boasts of a strong extended system of family and friends, who would be assumed to encourage screening practices. Moreover, the participants of our study did not significantly agree that “embarrassment or fear” of screening procedures hindered their participation. However, more females reported “fear of getting abnormal results” than males; this was seen as a fundamental barrier to screening (p < 0.005). Similar findings have been reported by Jillson, et al. [26]. Females who misconstrue screening procedures are more likely to dissuade fellow friends and family, and stigmatize screening practices which may have detrimental future effects. This trend accentuates the importance of education, as participants with a higher knowledge score were unlikely to believe that the screening procedure was “not safe” or “not effective”.

We also found evidence suggesting that males and adults with a university level education fared significantly better with respect to future intent towards CRC screening (p < 0.005). Similar results have also been reported by Guessous, et al. in his systematic review [19]. The above additive observations could possibly point towards a ‘lack of education and awareness’ as a major cause of non-adherence to screening recommendations. This suggests that if patients are explained about the severity of the disease, and the potential fatal complications, adherence to screening can be increased, which can easily be done by implementing mass awareness campaigns and creating means of easily accessible information to the urban population of Pakistan. Some of the answers to questions regarding barriers show optimism among the community as most people agreed on undergoing a colonoscopy if recommended by their doctor. Hence, a physician’s role is vital in removing barriers to screening participation for colorectal cancer in a community [27].

Despite our best efforts, there are still some limitations to our study. Firstly, our sample size is small and from a single city, which is not a representation of the public awareness needed in the entire country. Secondly, our participants were restricted to the urban dwellers of Karachi. Consequently, the results of this study cannot be extrapolated to rural settings where a lack of education, different cultural beliefs and a dearth of healthcare facilities may have strong influences on CRC screening practices. Therefore, we urge further study designs to be based in rural settings. Nonetheless, the findings of our study are vital in assessing the knowledge and perceptions regarding CRC and barriers to effective screening practices in a Pakistani population.

## Conclusions

It can be concluded that Pakistan needs a country-wide survey which can rectify the reported modifiable factors. These factors include barriers to uptake screening in rural and urban centres, and within people of all age groups and socio-economic statuses. Furthermore, we urge the government to set up inexpensive screening facilities and initiate awareness campaigns to prevent CRC from becoming a public health dilemma. Earlier diagnosis of the cancer can result in a better prognosis. In addition, our study's findings call upon the general practitioners of Pakistan to aim to dispel various fears and anxieties, counsel their patients and endorse good screening practices for a healthier Pakistan.
